# Tunable Protein Stabilization *In Vivo* Mediated by Shield-1 in Transgenic Medaka

**DOI:** 10.1371/journal.pone.0131252

**Published:** 2015-07-06

**Authors:** Alexander Froschauer, Lisa Kube, Alexandra Kegler, Christiane Rieger, Herwig O. Gutzeit

**Affiliations:** Institute of Zoology, Technische Universität Dresden, Dresden, Germany; National University of Singapore, SINGAPORE

## Abstract

Techniques for conditional gene or protein expression are important tools in developmental biology and in the analysis of physiology and disease. On the protein level, the tunable and reversible expression of proteins can be achieved by the fusion of the protein of interest to a destabilizing domain (DD). In the absence of its specific ligand (Shield-1), the protein is degraded by the proteasome. The DD-Shield system has proven to be an excellent tool to regulate the expression of proteins of interests in mammalian systems but has not been applied in teleosts like the medaka. We present the application of the DD-Shield technique in transgenic medaka and show the ubiquitous conditional expression throughout life. Shield-1 administration to the water leads to concentration-dependent induction of a YFP reporter gene in various organs and in spermatogonia at the cellular level.

## Introduction

The analysis of gene function in development and in adult organisms requires the controlled expression of proteins of interest. However, many genes have pleiotropic functions during embryogenesis and adulthood, hence the expression of a transgene may cause embryonic phenotypes that impede the analysis of later developmental stages. Therefore, several conditional expression systems have been developed to avoid embryonic phenotypes and to restrict the expression temporally and spatially [1]. Typically, two transgenes are used to control the expression of the gene of interest. The effector transgene defines the time and tissue of expression, activating or repressing the target transgene. In combination with transgenic methods to express fluorescent proteins in specific cell types, the observation of environmental or genetic effects is possible at the level of tissues or cells [2].

Activation of the target gene can also be achieved by a single transgene using the heat shock promoter for activation. Heat shock in zebrafish [3] and medaka [4] causes reliable expression of a transgene, but not all tissues have been tested and not all organs responded to this stress signal. In the medaka, the gonadal cells failed to activate the transgene upon heat shock treatment [4]. However, gonadal somatic cells and germ cells have been repeatedly labeled by fluorescent proteins in transgenic medaka [5–7]. Hence, the inefficient activation in these cells is seemingly not related to transgenesis but to a different response to heat shock.

The DD-Shield-System combines the simplicity of using a single transgene with the dose-dependent effect of Shield-1 to stabilize the protein of interest [8]. The authors isolated a double mutation (F36V, L106P) of the rapamycin-binding protein FKBP12 that confers a high level of instability to fusion partners of this protein domain and termed it the destabilizing domain (DD). The specific binding of the synthetic ligand Shield-1 to DD stabilizes the fusion protein that is otherwise degraded via the proteasome. Remarkably, this degradation is nearly complete in N-terminal fusions and can be fully restored by the addition of 1 μM Shield-1. This effect is concentration dependent and little if any side effects could be observed on the overall gene expression [9]. The system has been extensively tested in cell cultures [8], the protozoan *Entamoeba histolytica* [10], the flatworm *Caenorhabditis elegans* [11], in transgenic xenografts [12] and transgenic mice [13]. However, the DD-Shield system has not yet been applied in teleosts like the medaka (*Oryzias latipes*), which is a unique model organism in genetics, developmental biology, toxicology and carcinogenesis [14].

In principle, the fine tuning of expression using DD-Shield-1 can be achieved in every cell that expresses the fusion protein. For an *in vivo* treatment with any molecule, diffusion barriers like epithelia or the blood brain barrier may block or diminish the drug availability, change the kinetics of induction or may actively transport or degrade a drug like Shield-1. In this report we determined experimentally, whether the DD-Shield system is a practical approach for tunable protein expression in the medaka and whether all tissues including the gonads respond to the treatment.

## Materials and Methods

### Medaka stocks

*Oryzias latipes* strains d-rR.YHNI [15], FLFII [16] and FSI [17] were kept under standard conditions [18]. Hybrids of these strains are healthy and fertile and we refer to such hybrids as ‘Ola’ when specific features like the *leucophore free* locus [19] are not selected for in the breeding process.

This study was carried out according to the German regulations for animal welfare and approved by the Saxonian government (Landesdirektion Sachsen, 24–9168.11-8/2009-1).

### Vectors

Vectors for the Ac/Ds-transposon system [20] were kindly provided by Sergey Parinov, Temasek Life Sciences Laboratory, Singapore. For a nearly ubiquitous expression of a destabilized yellow fluorescent protein (DD-YFP) in medaka, the open reading frame of EGFP in pDS-Act1 [21] was replaced with the fusion ORF of pBMN-L106P-YFP [8] using *Nco*I and *Not*I. The resulting plasmid was termed pDS-actb-DD-YFP (S1 and S2 Files) and contains endogenous *actb* sequences of the medaka driving the DD-YFP expression.

### Transgenesis and identification of founders

Transgenic fish were generated by microinjection of transposase mRNA and plasmid pDS-actb-DD-YFP into 1-cell stage embryos of the strain d-rR.YHNI. The methodological details of the Ac/Ds system in medaka have been described earlier [21, 22]. Embryos were incubated in embryo culture medium at 26°C until hatching. Larvae were transferred to flow-through tanks and raised to maturity.

Founder individuals were then mated to each other or to non-transgenic fish. The individuals inheriting the transgene were identified either by the YFP expression in the offspring or by genotyping for the transgene in genomic DNA samples (F1 and later generations). Primers for the amplification of the transgene were *DD-Nar-Nco-F* (ggcGCCATGGGAGTGCAGGTGGAA, start codon of fusion ORF underlined) and *EGFP-C-REV* (ACGAACTCCAGCAGGACCAT, position 655–674 in *YFP*).

### Identification of integration sites

Genomic DNA was isolated from larvae or from tail fin tissue of adult fish. Sequences adjacent to the integration site were amplified by TAIL-PCR as described [21], sequenced and identified in the *ensembl* database using the BLAT/BLAST algorithm (http://www.ensembl.org/; medaka genome version 55.1i).

The Ensembl genomic sequences were used for primer design, matching sequences either on both sides of the integration site (wild-type allele) or on the transgene and the adjacent genomic region (transgenic allele). Individual fish were genotyped according to the respective integration sites in the sub-line (S1 Table). Primers matching the Ds-sites in the transgene were DS5-1 (CCGTTTACCGTTTTGTATATCCCG) or DS3-1 (CGATTACCGTATTTATCCCGTTCG).

### *In vivo* treatment and image analysis

Stock solutions (1–10 mM) of Shield-1 (kindly provided by Tom Wandless or Clontech’s #632188) were dissolved in absolute ethanol and stored at -20°C. Fish were treated by addition of stock solution directly to the water at 26°C. Up to 6 larvae were treated in a total volume of 3 ml tap water supplemented with 0.03% (w/v) sea salt, adult fish were treated in 20 ml conditioned tap water per individual. The fish were fed every second day and the water was changed after feeding.

For image analysis the fish were sedated with 100 mg/l MS-222 (E10521, Sigma) in tap water and observed under a fluorescence stereomicroscope (Leica MZ10F) equipped with a YFP filter set (510/20 nm excitation, 560/40 nm emission). Photographs were taken with a monochrome digital camera (Zeiss MRm) and processed using Adobe Photoshop CS2 (Adobe Systems Corporation). For quantification of fluorescence in larvae, the area of the whole specimen was defined in the bright field image and the intensity of fluorescence was determined as the mean grey value of this area in the fluorescence image. Figures show original photographs or colored overlays of bright field and fluorescence.

### Western blot

After individual treatment, brain, liver and testis were sonicated in 1 ml RIPA buffer containing protease inhibitors (cOmplete ULTRA, Roche, Germany) per 50 mg tissue. Each sample was adjusted to the same protein concentration before Western blotting of a 10% SDS-PAGE gel on a PVDF membrane. Rabbit polyclonal anti-GFP (1:5,000; ab290, abcam, Cambridge) and HRP-conjugated anti-rabbit (1:7,500; #111-035-144; dianova, Germany) antibodies were used at the indicated dilutions before immunodetection with chemiluminescence (Luminata forte Western HRP substrate, Merck, Germany).

### Flow cytometry

Primary testis cells were isolated and analyzed as described [17]. Shield-1 was present during the isolation procedure in the same concentration as in the *in vivo* treatment. DNA content (Hoechst 33342; 5 μg/ml; Life Technologies) and YFP fluorescence were analyzed using a CyFlow Space flow cytometer (Partec, Germany) at wavelengths of 375/455 nm (Hoechst stain) and 488/527 nm (GFP and YFP).

## Results

### Functionality of the transgene

Sequences flanking the coding region of the cytoplasmic actin gene (*actb;* promoter, exon 1, intron 1 and 3’UTR) led to a nearly ubiquitous expression of EGFP in transgenic medaka [21]. The plasmid pDS-actb-DD-YFP (S1 File) contains these endogenous regulatory elements driving the expression of the fusion protein DD-YFP [8]. To assay conditional expression, HEK-293T cells were transfected with pDS-actin-DD-YFP, cultured for 24 hours and treated with Shield-1. The HEK-293T cells showed a dose-dependent increase of yellow fluorescence after Shield-1 treatment (0.1, 0.8 and 1 μM) and over time (1, 5, 24 h) with no visible fluorescence in untreated cells (data not shown).

### Establishment of transgenic lines

The microinjection of pDS-actin-DD-YFP and transposase RNA in embryos of the 1- or 2-cell stage led to a typical mosaic transgenesis pattern that was visible after treatment of freshly hatched larvae of the founder generation with 0.8 μM Shield-1 (not shown). Two founder males showed particularly strong mosaic fluorescence patterns but only one was successfully mated. The germline transmission rate of this founder male was not determined, but individuals of the F1 generation transmitted the transgene to over 80% of their progeny. Such a high rate of transmission is indicative for multiple integrations. Seven different integration sites of the transgene were determined by TAIL-PCR (S1 Table).

### Establishment of line Ola-Tg(actin-DD-YFP)13

For the experiments we chose individuals with identical transgenic status to exclude position effects (S2 Table). One individual fish being hemizygous for a single integration of the transgene could be identified in the F3 generation. The sub-line Ola-Tg(actin-DD-YFP)13 was established from this male that inherited only one out of seven possible integrations. Individuals of the F4 (hemizygous for the transgene) to the F6 generation (homozygous) were used for the experiments. For Western blot analysis, 24 individual fish of Ola-Tg(actin-DD-YFP)13 of the F4 generation were genotyped for the presence of the transgene on chromosome 13. The 14 positive individuals were considered to be hemizygous since the remaining 10 individuals had no transgene in the genome. For flow cytometry analysis, hemizygous fish of Ola-Tg(actin-DD-YFP)13 of the F5 generation were used.

### Induction of YFP in fry and embryos

#### Induction in fry

**Fig 1:**
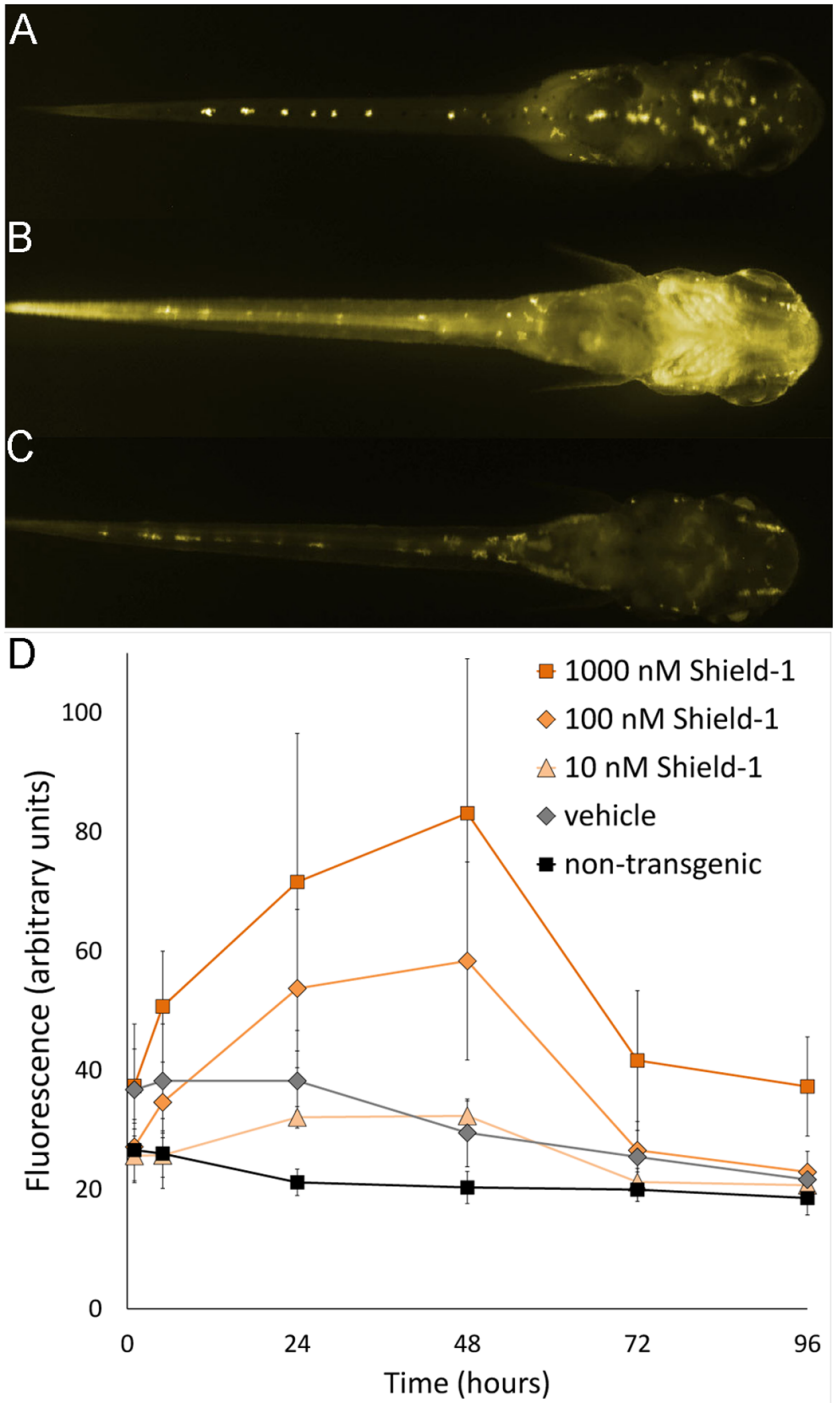
Kinetics of YFP induction in fry. Transgenic fries were treated with the indicated concentrations of Shield-1 or 0.1% ethanol (vehicle). After 48 hours, the water was exchanged and the imaging was continued up to 72 and 96 hours to monitor the reversibility of the induction. (A-C) Fluorescence images of a representative individual treated with 100 nM Shield-1; dorsal view, head to the right. The faint fluorescence seen in transgenic individuals after 1 hour (A) increases to maximum after 48 hours (B) and decreases to background level after 96 hours of the experiment (C). (D) Fluorescence values (area fluorescence, arbitrary units) after treatment of fry with the indicated concentrations of Shield-1. Data points of three individuals are shown for the vehicle control, 5–6 individuals for the other groups (mean with standard deviation). The images were taken over a period of 96hours with an exposure time of 10 seconds. The monochrome images in A-C were colored for clarity.

Ten freshly hatched fry (F4 generation) per concentration were treated with Shield-1. An induction of YFP could be observed within a few hours in transgenic fry (Fig 1). Each individual was genotyped after the experiment and the data of the transgenics were used for the analysis of leakiness and the kinetics of induction *in vivo*. The presence of autofluorescent yolk and leucophores defines the basal level of fluorescence observed in non-transgenic fish and transgenics, respectively (Fig 1C).

#### Kinetics and dose dependency

For the analysis of the induction kinetics, 10 individuals per concentration were treated with 10, 100 or 1,000 nM Shield-1, respectively. The fluorescence was analyzed after 1, 5, 24 and 48 hours. After 48 hours, the inducer Shield-1 was removed to test the reversibility of the induction after 72 and 96 hours (Fig 1A-1C). The fluorescence increased over time after a single administration of Shield-1 and the induction was concentration dependent (Fig 1D). Shield-1 led to a strong increase in fluorescence after 5 and 24 hours using 100 nM and 1,000 nM inducer, respectively. The fluorescence values were significantly different from the vehicle control (Fig 1D and S2 Fig). The induction reached a dose-dependent, steady-state level after approximately 24 hrs. After withdrawal of Shield-1 at 48 hours the fluorescence decreased to basal levels within the following 48 hours in the larvae treated with 10 or 100 nM Shield-1. Treatment with 1,000 nM Shield-1 caused a strong and significant induction as early as 1 hour, did not reach a steady state and failed to be fully reversible within 48 hours (Fig 1D).

#### Leakiness

**Fig 2:**
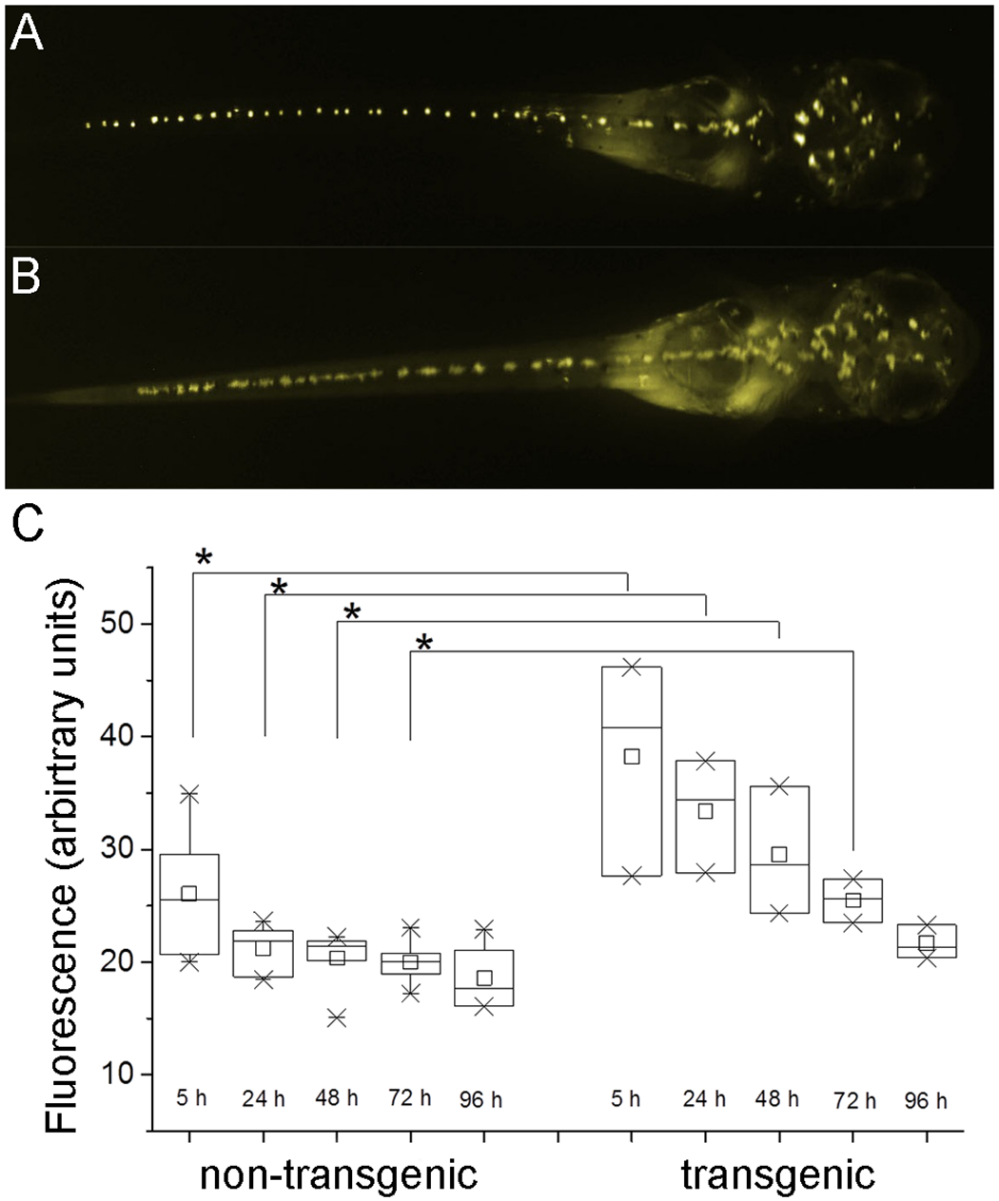
Background fluorescence in transgenic fry of Ola-Tg(actin-DD-YFP)13. The fluorescence of hemizygous transgenic individuals treated with 0.1% ethanol and non-transgenic siblings was analyzed. (A) non-transgenic fry, (B) transgenic, non-induced fry. Monochrome images were colored for clarity, head to the right. (C) Box plot (25–75%), average (open square) and mean (line) of the fluorescence (arbitrary units) are shown. The values are normally distributed (Kolmogorow-Smirnov-test) in all samples and are significantly higher (asterisks, p≤0.05, ANOVA) for transgenics compared to non-transgenic fish within the first 72 hours of the experiment.

Non-induced individuals of the line Ola-Tg(actin-DD-YFP)13 (Fig 1D) showed a weak fluorescence that is merely detectable by eye in the tail and somites (Fig 2A & 2B) but could be quantified by image analysis (Fig 2C & S2 Fig). The transgenic individuals show twice the area fluorescence (arbitrary units) than non-transgenics at the beginning of the analysis. Two factors contribute to the observed decrease of fluorescence. First, the yolk mass is autofluorescent and is metabolically degraded over time in both groups. Second, the yolk is enclosed by epithelia of the gut and epidermis. These epithelia are fluorescent in transgenic fry, hence leading to a disproportional contribution to the total area fluorescence within the first 48 hours (Fig 2C). At the end of the experiment the fluorescence values of the two groups are converging.

#### Induction in embryos

Shield-1 treatment of embryos led to an induction of fluorescence in transgenic embryos (S1 Fig). However, the induction and degradation of YFP was observed later in the embryo than in the fry. Probably the diffusion of Shield-1 through the chorion is leading to a delayed kinetics of uptake and removal as compared to the fast kinetics in hatchling and fry, respectively.

### Induction in adult medaka

**Fig 3:**
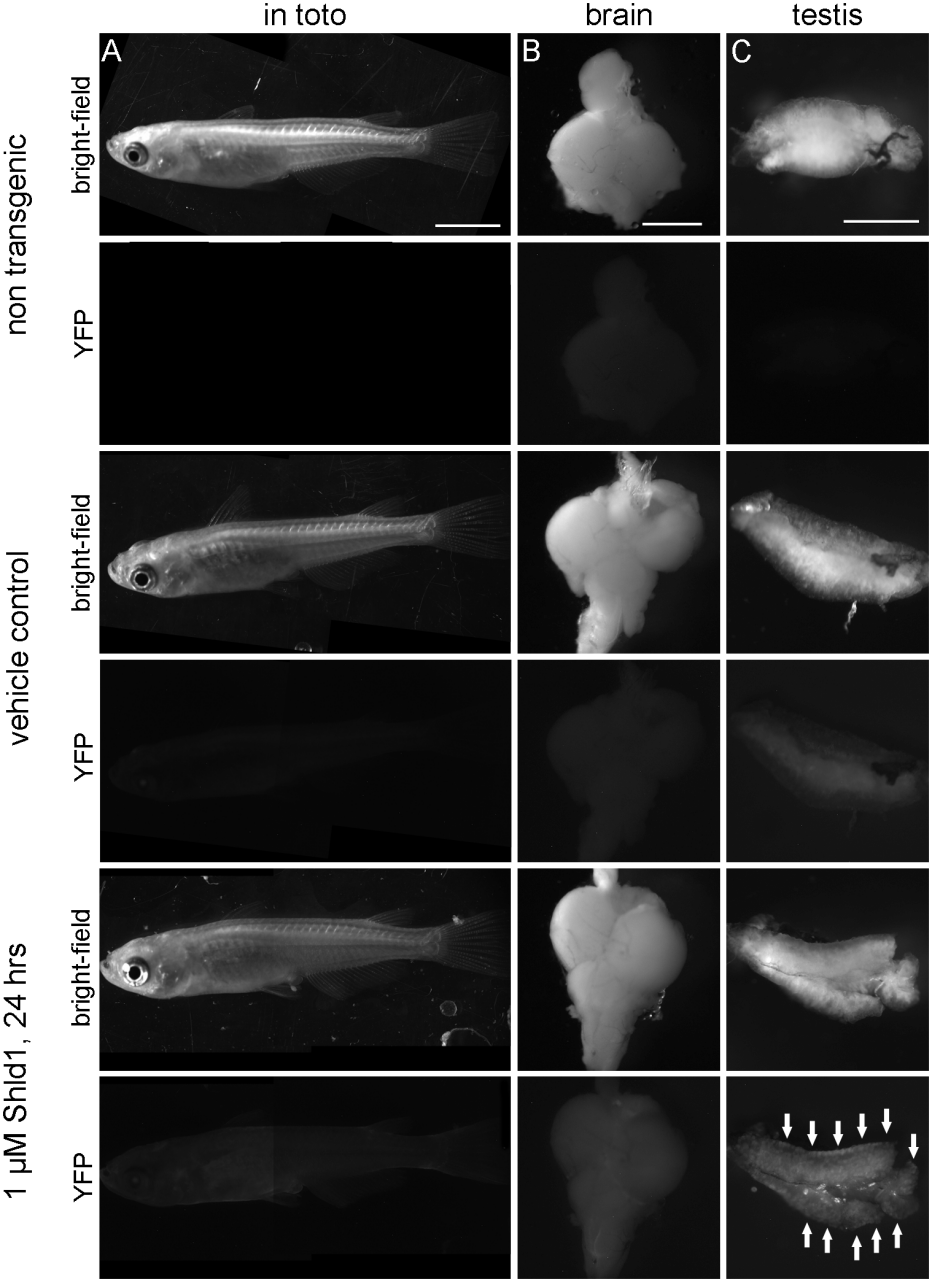
Induction of YFP in adult medaka. The fluorescence of non-transgenic adult fish and of transgenic individuals of Ola-Tg(actin-DD-YFP)13 was photographed after the indicated treatment with vehicle only (0.1% ethanol) or 1 μM Shield-1, respectively. (A) Adult females are shown as combinations of 2 overlapping photographs *in toto*; exposure time: 13 sec, scale bar: 5 mm. (B) Brains of the same individuals as in A; exposure time: 21 sec, scale bar: 1 mm. (C) Testes of adult males show the strongest fluorescence at the outer area at the end of the tubules (arrows); exposure time: 13 sec, scale bar 1 mm.

Adult male and female individuals were treated with different concentrations of Shield-1 and analyzed after 24 hours. The induced fluorescence was visible *in toto*, in brain and in gonads (Fig 3 and S3 Fig). The induction of YFP in brain (Fig 3B) shows that Shield-1 effectively passes the blood brain barrier. Other tissues like gills, gut, liver, kidney, spleen and heart also responded to the treatment (not shown). We did not compare the fluorescence levels between different organs due to their differences in cellular composition, thickness and autofluorescence, but the induction was dose dependent in all organs.

The stabilization of the DD-YFP fusion protein was confirmed in an immunoblot. Adult fish were treated with different concentrations of Shield-1 and protein extracts were isolated after different time points (S4 Fig). The fusion protein was detected with a polyclonal anti-YFP antibody in brain and testis extracts but the strong unspecific signals of yolk proteins masked the expected protein band in extracts of the ovary (not shown). The expected signal at 40 kD is only present in the soluble proteins of transgenic fish proofing its identity. A single administration of Shield-1 leads to a faithful stabilization for at least 48 hours. From these data, no conclusions can be drawn about the kinetics of protein protection, but YFP is stabilized after induction and the level of protein is stable for 48 hours after treatment.

**Fig 4:**
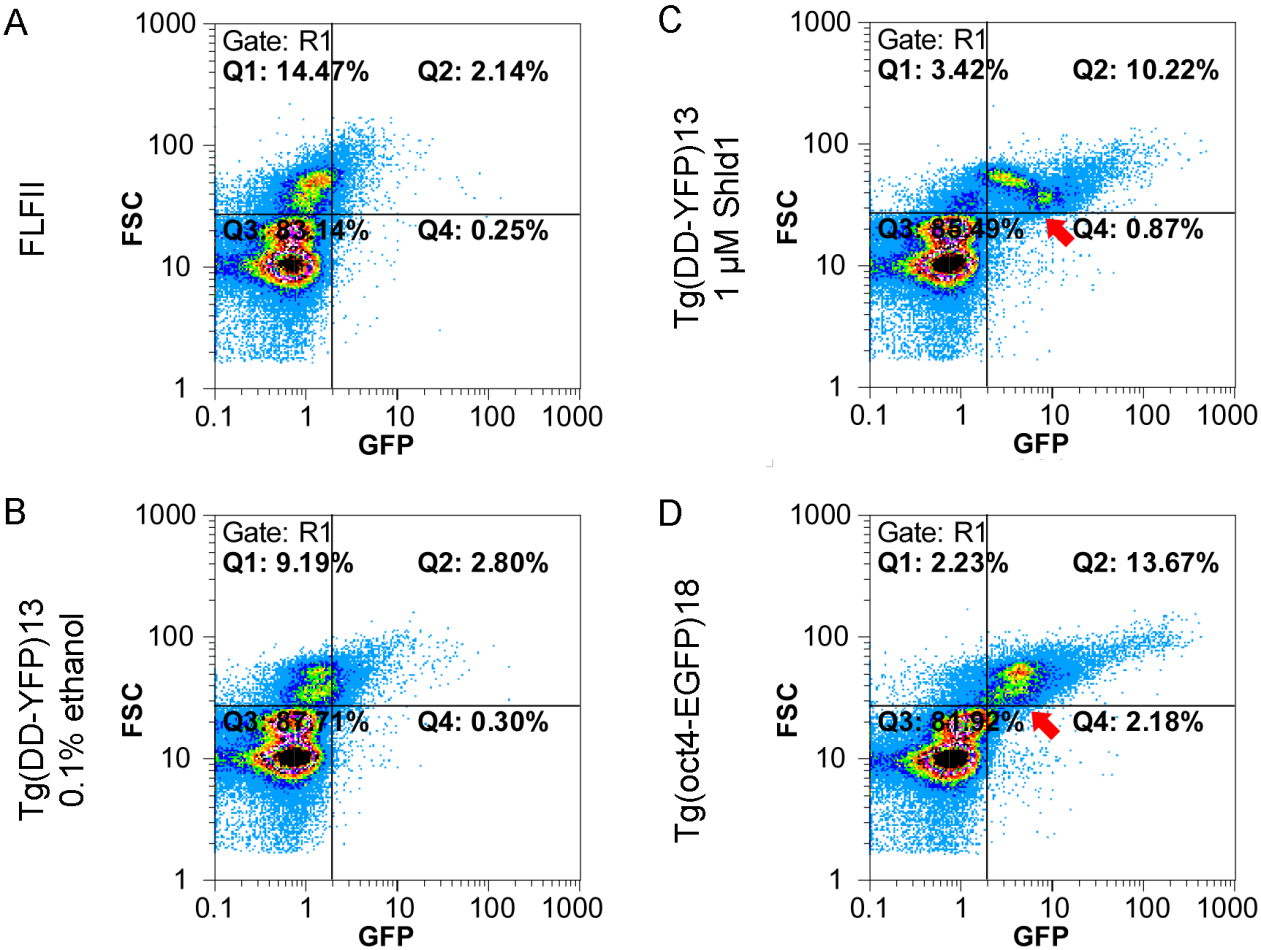
Induced fluorescence of testis cells analyzed by flow cytometry. Primary testis cells of hemizygous Ola-Tg(DD-YFP)13 were analyzed for size (FSC), fluorescence (GFP) and DNA content (Gate R1; Hoechst staining, not shown). The quadrants Q1 and Q2 contain cells with a DNA content of 2C to 4C, i.e. somatic cells and germ cells prior to meiosis. The plots show the data of 129–154,000 single cells. (A) The fluorescence in a sample of a non-transgenic FLFII and was plotted against the cell size. (B) In the non-induced control virtually no additional fluorescence is observed in Q2 compared to FLFII. (C) Treatment with 1 μM Shield-1 causes a strong induction of fluorescence in the largest cells that comprise the stem cell fraction. Absolute fluorescence and number of cells in Q2 are increased. (D) For comparison, the GFP-positive stem cells in FLF-Tg(oct4-EGFP)18 are shown [17]. In this line, the fluorescence decreases during diffentiation (arrow). In contrast, a cell fraction of similar size has an elevated fluorescence after induction (arrow in C). These cells represent the mitotically active type B spermatogonia that actively transcribe the *actb*-driven DD-YFP but not the *oct4*-driven EGFP.

The gonadal stem cells of the medaka are located in the outer tubular compartment of the testis and in the cradles of the ovary [23, 24]. Remarkably, these regions showed an inducible YFP fluorescence after *in vivo* treatment (Fig 3C and S3 Fig). For the testis, we quantified the induced fluorescence by flow cytometry (Fig 4). Primary testis cells were isolated from transgenic fish after 24 hours *in vivo* treatment. For comparison, cells of non-transgenic FLFII (Fig 4A) and cells of an *oct4*-EGFP reporter line that labels the stem cell fraction of the testis (Fig 4D) were isolated. Identical gates and filters were used for the analysis; the quadrants indicate the background fluorescence (Q1, Q3) on the horizonzal axis and the cells size of the somatic cells and pre-meiotic germ cells (Q1, Q2).

In primary testis cells of Ola-Tg(DD-YFP)13 a minimal, if any, increase of fluorescence (Q2) is observed after ethanol treatment compared to the non-transgenic cells (Fig 4A & 4B). A strong increase in fluorescence is induced by *in vivo* treatment with 1 μM Shield-1 for 24 hours (Fig 4C). The percentages of cells in Q1 and Q2 are similar those of FLF-Tg(oct4-EGFP)18 cells (Fig 4D). The stem cells of this transgenic line are found in Q2 and show the highest transgene expression in the spermatogonia [17]. The fluorescence of these cells decreases during mitotic divisions and differentiation. In contrast, a strong induction of YFP is observed in the smallest cells of the pre-meiotic cell population in Ola-Tg(DD-YFP)13 (Fig 4C). These cells belong to the mitotically active type B spermatogonia. Post-meiotic cells do not show an elevated fluorescence in all samples.

## Discussion

The DD-Shield system is designed for the expression of a fusion protein that is efficiently degraded in the absence of Shield-1. We have chosen the reliable promoter of the *actb* gene [21] to express a DD-YFP fusion protein. This promoter is known to drive a nearly ubiquitous expression with the exception of red blood cells and probably the highly autofluorescent melanophores [21]. A certain variability of the expression level among cells and tissues still allowed monitoring the kinetics and dose dependency of the DD-Shield system in the medaka *in vivo*.

At concentrations ranging from 10 to 1,000 nM Shield-1 we observed a concentration dependent level of induction. A low level of leakiness could be quantified in untreated individuals probably caused by the strong *actb* promoter. However, the efficient degradation of YFP after withdrawal of 1,000 nM Shield-1 indicates highly active proteasomal degradation in medaka. This degradation capability should minimize any effects of the transgene when weaker promoters are used. The pharmacokinetics of Shield-1 was not analyzed with respect to absorption, metabolism and excretion. However, the *in vivo* treatment with 10–100 nM Shield-1 leads to a faithful and revertible induction of the transgene in medaka.

The administration of Shield-1 to the water is the most convenient way to expose fish from early embryonic stages to adults. We did not test the alternative ligand AquaShield-1 with optimized aqueous solubility that recently became available. The induction kinetics of Shield-1 *in vivo* is fast and dose dependent in medaka. The fact that gills and the gut show the strongest induction in larvae could be due to the direct contact with the inducer or the high DD-YFP expression level in these epithelia. A stronger fluorescence in the gut has already been observed for non-inducible EGFP using the same promoter sequences [21]. Shield-1 crosses the blood brain barrier efficiently. It is also active in the stem cells of the testis. This is remarkable since gonadal cells failed to respond to heat shock treatment in earlier experiments [4]. After treatment with Shield-1, the fluorescence pattern of primary testis cells resembles the pattern of oct4-EGFP expressing cells in flow cytometry. Undifferentiated spermatogonia divide slowly but proliferate actively during spermatogenesis. Oct4-EGFP is expressed in the spermatogonia of the medaka and the stem cell-specific fluorescence decreases during mitotic division and differentiation of the germ cells [17]. In contrast to oct4-EGFP expressing cells, the differentiating fraction of actb-DD-YFP cells has an increased fluorescence after induction with Shield-1 (Fig 4C and 4D). Cytoplasmic actin is an important factor for spindle formation in mitosis and meiosis, mostly studied on the level of protein dynamics [25]. Conceivably, an elevated expression of actin precedes this proliferative burst in the respective cell fraction and the actb-driven DD-YFP expression is simultaneously activated in our experimental setting. Irrespective of this specific pattern, DD-Shield-1 allows the targeting of germ cells in future experiments using conditional protein expression.

The use of a single transgene is convenient since transgenesis and stock management is facilitated. It might also be of interest to fuse the destabilizing domain to an endogenous gene, resulting in a conditional knock down/ knock out of the target gene. This has been reported for human cells *in vitro*, in which the first exon of TCOF1 was replaced by a DD-tagged sequence [26]. Park et al. achieved this genomic engineering by CRISPR/Cas9-mediated homologous recombination and a donor template coding for a resistance cassette and the DD-fusion sequence [27]. In a similar approach, Chen et al. (2013) introduced a hygromycin resistance cassette to the ben-1 locus in *Caenorhabditis elegans* and isolated resistant individuals of the F2 generation [28]. These selection procedures make an estimation of the efficacy difficult for teleosts, but CRISPR-Cas9 has been already used to introduce sequence modifications in target genes of zebrafish [29] and medaka [30]. Therefore, the targeted knock-in of the destabilizing domain to the gene of interest *in vivo* is principally possible. However, Shield-1 is an expensive inducer when adult individuals are to be treated. As an alternative, the destabilizing domain derived of the bacterial dihydrofolate reductase [31] could be used. Its inducing molecule trimethoprim is less expensive and is an approved drug crossing the blood brain barrier when administered via the drinking water to rats [32]. Therefore, in the case of establishment in medaka, two excellent systems would be available in the future.

From our data we can conclude that the DD-Shield system in medaka is a valuable tool for conditional protein expression *in vivo*. Its major advantage is the induction on the level of the protein showing a fast and reversible response that allows the fine tuning of active protein levels and exceeds the control by induced transcription only.

## Supporting Information

S1 File Graphic map of pDS-actb-DD-YFP. The sequence was combined from databases (vector) and sequencing results during cloning. The map was created with Serial cloner version 2.6.1.
(TIF)

S2 File Sequence of pDS-actb-DD-YFP in GenBank format. The sequence was combined from databases (vector) and sequencing results during cloning. The file was created with Serial cloner version 2.6.1.
(TXT)

S1 Table Integration sites of the transgene and primers for genotyping. The sub-lines of Ola-Tg(actin-DD-YFP) were named by the chromosome carrying the integration, e.g. Ola-Tg(actin-DD-YFP)13. The chromosome cannot be identified in the case of repetitive sequences (Repeat 4).
(PDF)

S2 Table Genotypes of individual fish analyzed in this work. Individuals #1–19 were of the F2 generation, #20–23 of F3. Only individual #21 had a single integration (C13) and was used for further breeding and analysis of embryos. (*) marks the female individuals used for induction in S3 Fig.
(PDF)

S1 Fig Induction of YFP in embryos. Embryos homozygous for the integration of DD-YFP on chromosome 13 (F6) were treated at blastula stage with vehicle only (A) or 1 μM Shield-1 (B, C) and photographed every 24 hours; dorsal view, head to the left. Withdrawal of Shield-1 after 48 hours led to a reduction of fluorescence (B’). Images were taken with fixed exposition time of 11 s (1 dpf), 4 s (2 dpf) and 500 ms (3 & 4 dpf), respectively. The fluorescence in row A (1 dpf– 4 dpf) is merely visible. Dpf: days post fertilization.
(TIF)

S2 Fig Statistical analysis of fluorescence values after 24 and 48 hours of treatment. Box plots (25–75%), average (open square) and mean (line) of the fluorescence (arbitrary units) are shown for each sample. A significant increase of fluorescence (asterisks, p≤0.05, ANOVA) can be observed between the vehicle control and the 100 nM and 1 μM samples, respectively. 10 nM Shield-1 did not lead to a significant increase in fluorescence as compared with the vehicle control, but the vehicle control is significantly different from the non-transgenic sample.
(TIF)

S3 Fig Photographs of adult medaka and organs after induction with Shield-1. A non-transgenic individual, non-induced control (0.1% ethanol) and treatment with 10 nM or 1 μM Shield-1 for 24 hours are shown. Adult females with integrations of the transgene on chromosomes 15 and 19 (Table S4) were treated as indicated. The fluorescence (YFP channel) was photographed with a monochrome camera at a constant exposure time of 13 sec.
(TIF)

S4 Fig Western blot and immunodetection of YFP in brain and testis. Total protein of male brain (A) and testis (B) was isolated after the indicated treatment. A non-transgenic control fish does not show the expected signal at approximately 40 kD (white asterisks). The transgenic fish show signals at different intensities: the vehicle control (0.1% ethanol) is not negative but the induced fish show a clear stabilization of DD-YFP fusion proteins after 24, 48 and 96 hours. The polyclonal anti-GFP antibody has a high cross reactivity to YFP and other medaka proteins, this served as loading control. The HRP-coupled secondary antibody used for chemiluminescence detection did not cross-react (not shown).
(TIF)
