# Supplementary figures and images for: Tunable Protein Stabilization In Vivo Mediated by Shield-1 in Transgenic Medaka
Source: PLoS One. 2015 Jul 6;10(7):e0131252. doi: 10.1371/journal.pone.0131252 (PMC4493054; doi:10.1371/journal.pone.0131252)

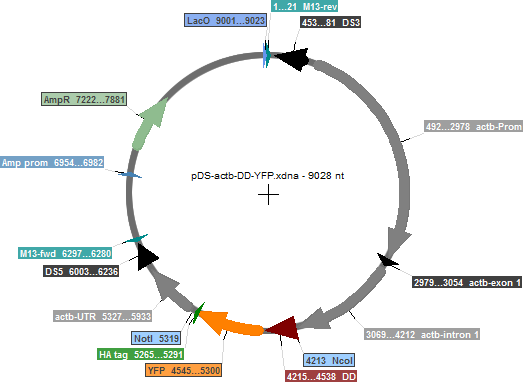

Supplement: S1 File — The sequence was combined from databases (vector) and sequencing results during cloning. The map was created with Serial cloner version 2.6.1. (TIF) [file pone.0131252.s001.tif]

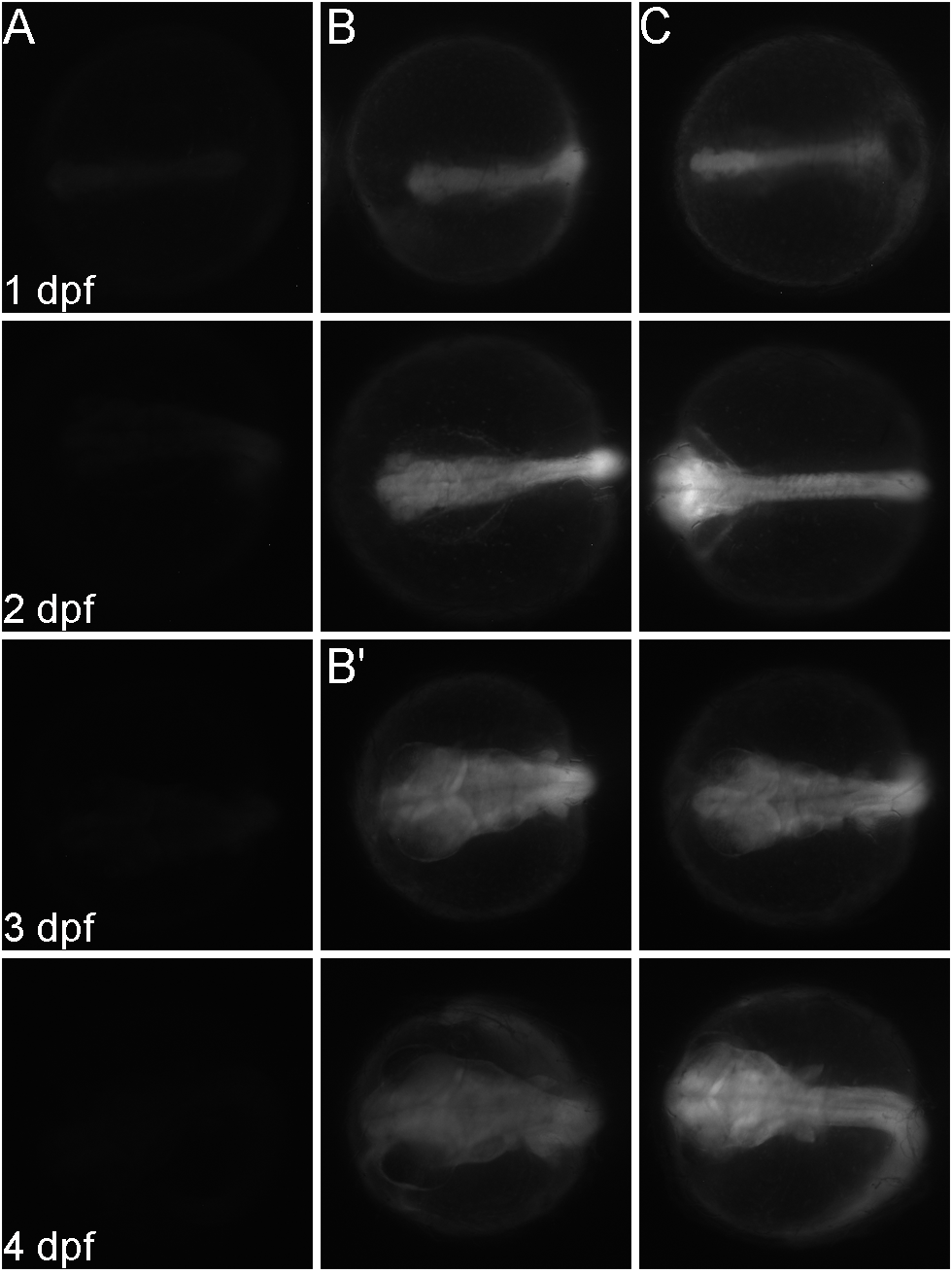

Supplement: S1 Fig — Embryos homozygous for the integration of DD-YFP on chromosome 13 (F6) were treated at blastula stage with vehicle only (A) or 1 μM Shield-1 (B, C) and photographed every 24 hours; dorsal view, head to the left. Withdrawal of Shield-1 after 48 hours led to a reduction of fluorescence (B’). Images were taken with fixed exposition time of 11 s (1 dpf), 4 s (2 dpf) and 500 ms (3 & 4 dpf), respectively. The fluorescence in row A (1 dpf– 4 dpf) is merely visible. Dpf: days post fertilization. (TIF) [file pone.0131252.s005.tif]

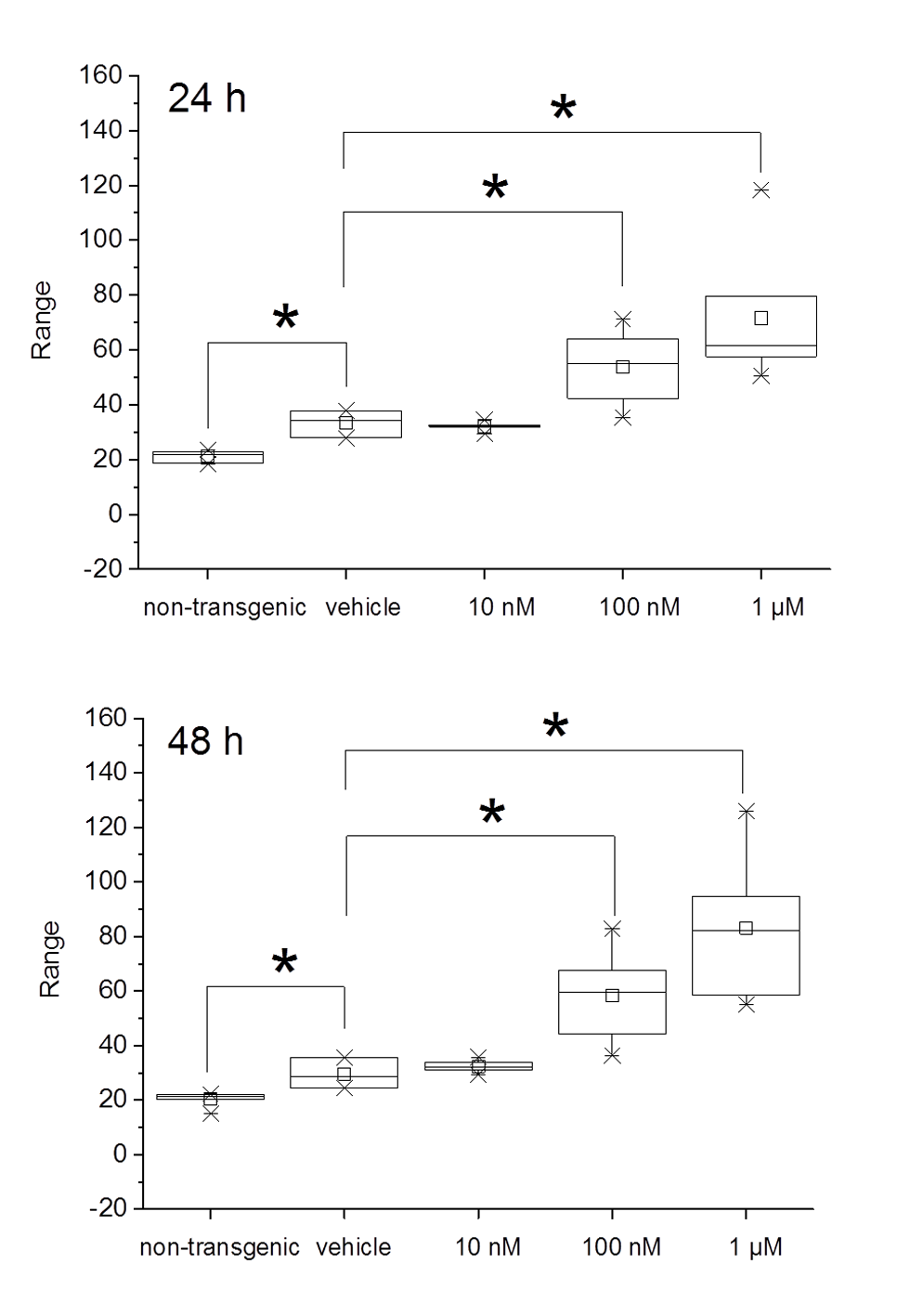

Supplement: S2 Fig — Box plots (25–75%), average (open square) and mean (line) of the fluorescence (arbitrary units) are shown for each sample. A significant increase of fluorescence (asterisks, p≤0.05, ANOVA) can be observed between the vehicle control and the 100 nM and 1 μM samples, respectively. 10 nM Shield-1 did not lead to a significant increase in fluorescence as compared with the vehicle control, but the vehicle control is significantly different from the non-transgenic sample. (TIF) [file pone.0131252.s006.tif]

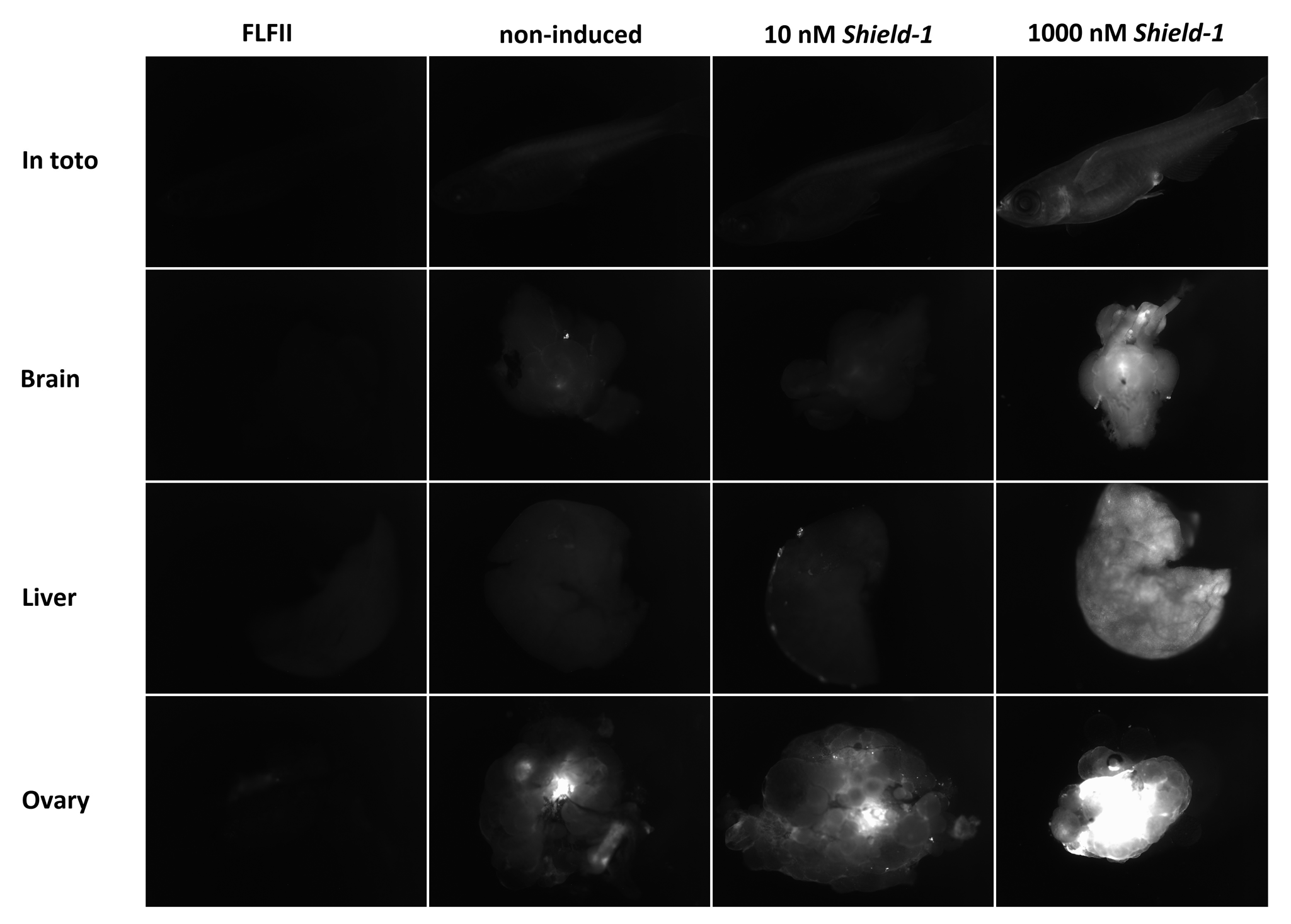

Supplement: S3 Fig — A non-transgenic individual, non-induced control (0.1% ethanol) and treatment with 10 nM or 1 μM Shield-1 for 24 hours are shown. Adult females with integrations of the transgene on chromosomes 15 and 19 (Table S4) were treated as indicated. The fluorescence (YFP channel) was photographed with a monochrome camera at a constant exposure time of 13 sec. (TIF) [file pone.0131252.s007.tif]

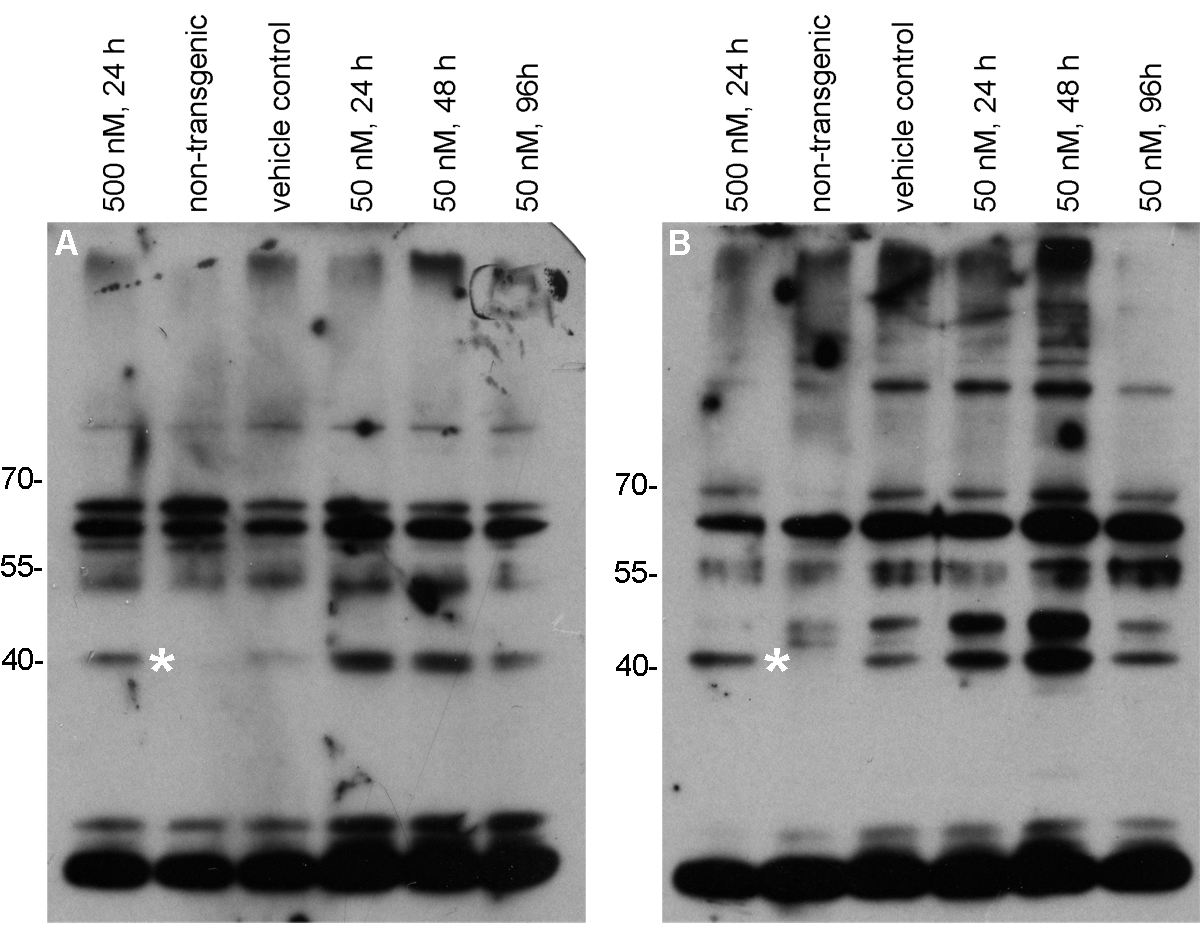

Supplement: S4 Fig — Total protein of male brain (A) and testis (B) was isolated after the indicated treatment. A non-transgenic control fish does not show the expected signal at approximately 40 kD (white asterisks). The transgenic fish show signals at different intensities: the vehicle control (0.1% ethanol) is not negative but the induced fish show a clear stabilization of DD-YFP fusion proteins after 24, 48 and 96 hours. The polyclonal anti-GFP antibody has a high cross reactivity to YFP and other medaka proteins, this served as loading control. The HRP-coupled secondary antibody used for chemiluminescence detection did not cross-react (not shown). (TIF) [file pone.0131252.s008.tif]
