# Supplementary material for: Tunable Protein Stabilization In Vivo Mediated by Shield-1 in Transgenic Medaka
Source: PLoS One. 2015 Jul 6;10(7):e0131252. doi: 10.1371/journal.pone.0131252 (PMC4493054; doi:10.1371/journal.pone.0131252)
Supplement: S1 Table — The sub-lines of Ola-Tg(actin-DD-YFP) were named by the chromosome carrying the integration, e.g. Ola-Tg(actin-DD-YFP)13. The chromosome cannot be identified in the case of repetitive sequences (Repeat 4). (PDF) [file pone.0131252.s003.pdf]

S3 Table: Integration sites of the transgene and primers for genotyping.

The sub-lines of Ola-Tg(actin-DD-YFP) were named by the chromosome carrying the integration, e.g. Ola-Tg(actin-DD-YFP)13. The chromosome cannot be identified in the case of repetitive sequences (Repeat 4).

Integration site (Chromosome: Mb)	Forward (FOR)	Reverse (REV)	Combination for transgene
**8: 10,044.60**	TCTGATGCCAGCATGCTGTC	CATAATCAAGCCTGGCAGTG	REV, DS5-1
**13: 24,192.94**	TGTACTCACTGTAGATGTGG	TTTGCGAGTCAGCTTACTGC	REV, DS3-1
**14: 10,746.85**	GCTTCAACATCTGTCCCTGG	CTTCAGTCCTGCATAGGTTGA	FOR, DS3-1
**15: 11,274.07**	TACCACTGAGCGAGCTGTT	TCATAATGAAGACCACTAGG	REV, DS5-1
**19: 16,131.20**	GAGAGATGCAATTCACAAGC	CGATCTTCGCGAGCTATGCT	FOR, DS3-1
**21: 17,218.32**	TTGCTCACTGGCTAGGATTG	ACTGAGTCCTCCGGTCCAAA	FOR, DS3-1
**Repeat 4**	GAAATGGCTGAACTGTACTGG	n.a.	FOR, DS3-1
