# Supplementary material for: Tunable Protein Stabilization In Vivo Mediated by Shield-1 in Transgenic Medaka
Source: PLoS One. 2015 Jul 6;10(7):e0131252. doi: 10.1371/journal.pone.0131252 (PMC4493054; doi:10.1371/journal.pone.0131252)
Supplement: S2 Table — Individuals #1–19 were of the F2 generation, #20–23 of F3. Only individual #21 had a single integration (C13) and was used for further breeding and analysis of embryos. (*) marks the female individuals used for induction in S3 Fig. (PDF) [file pone.0131252.s004.pdf]

S4 Table: Genotypes of individual fish analyzed in this work.

Individuals #1-19 were of the F2 generation, #20-23 of the F3 generation. Only individual #21 had a single integration (C13) and was used for further breeding and analysis of embryos. (*) marks the female individuals used for induction in Figure S7.

Individual	C8	C13	C14	C15	C19	C21	Repeat 4
#1	-	-	-	+	+	+	+
#2	-	-	-	-	+	+	-
#3	+	-	+	+	+	+	-
#4	-	-	-	-	-	+	+
#5	-	+	+	-	-	+	-
#6	+	-	-	-	+	+	-
#7	+	+	+	+	+	+	+
#8	+	-	-	-	+	-	+
#9	+	+	-	+	+	-	-
#10	-	-	-	+	+	-	-
#11	-	-	-	+	+	-	+
#12	+	-	-	+	-	+	-
#13	+	-	-	+	-	+	-
#14 *	-	-	-	+	+	-	-
#15	+	+	+	+	-	+	+
#16	-	-	-	+	+	-	+
#17 *	-	-	-	+	+	-	-
#18 *	-	-	-	+	+	-	-
#19	-	+	-	+	-	+	-
#20	-	+	-	-	-	+	-
#21	-	+	-	-	-	-	-
#22	-	-	-	-	+	+	-
#23	-	-	-	+	-	+	-
